# Dynamic Functional Synchronization Profiles in Autism Differ by Spatial Scale and Along Hierarchical Cortical Gradients

**DOI:** 10.1016/j.bpsgos.2026.100787

**Published:** 2026-07-08

**Authors:** Benedikt P. Fuhr, Yonatan Sanz Perl, Ines Severino, Morten L. Kringelbach, Gustavo Deco, Henricus G. Ruhé, Michael V. Lombardo

**Affiliations:** a Laboratory for Autism and Neurodevelopmental Disorders, Center for Neuroscience and Cognitive Systems, Istituto Italiano di Tecnologia, Rovereto, Italy; b Center for Mind/Brain Sciences, University of Trento, Rovereto, Italy; c Center for Brain and Cognition, Computational Neuroscience Group, Faculty of Medicine and Life Sciences, Universitat Pompeu Fabra, Barcelona, Spain; d Institució Catalana de la Recerca i Estudis Avançats (ICREA), Barcelona, Spain; e Centre for Eudaimonia and Human Flourishing, Linacre College, University of Oxford, Oxford, United Kingdom; f International Centre for Flourishing, Universities of Oxford (United Kingdom), Aarhus (Denmark), and Pompeu Fabra (Spain); g Department of Clinical Medicine, Aarhus University, Aarhus, Denmark; h Department of Psychiatry, University of Oxford, Oxford, United Kingdom; i Department of Psychiatry, Radboudumc, Nijmegen, the Netherlands; j Donders Institute for Brain, Cognition and Behavior, Radboud University, Nijmegen, the Netherlands

**Keywords:** Autism, Brain dynamics, Cortical gradients, Functional connectivity, Imaging, Resting-state fMRI

## Abstract

**Background:**

Prevailing theories propose that autism is characterized by local cortical overconnectivity and long-range underconnectivity, but empirical evidence remains mixed.

**Methods:**

Here, we applied the turbulence dynamics framework to the ABIDE (Autism Brain Imaging Data Exchange) dataset (*N* = 1009) to examine how functional synchronization profiles dynamically change over time and across the cortex over different spatial scales.

**Results:**

Autistic individuals showed increased short-range and reduced long-range functional synchronization variability over time, as well as reduced synchronization strength across all spatial scales. Synchronization also decayed more rapidly with distance and exerted weaker influence across scales in autism. These distance-specific alterations suggest that local hyperconnectivity may be associated with turbulent synchronization dynamics that fail to propagate coherently across the cortex, resulting in an overly rigid brain organization at longer distances. Mapping these effects onto the sensorimotor–association cortical gradient revealed increased variability in sensorimotor regions and decreased variability in the association cortex.

**Conclusions:**

Together, we found evidence of disturbances in functional synchronization dynamics at different spatial scales and along hierarchical brain gradients in autistic individuals. These results consolidate ideas about dynamic functional connectomic organization in autism and situate these alterations along hierarchical brain gradients that are closely linked to neurodevelopmental processes.

## Plain Language Summary

Autism is characterized by atypical neuronal connectivity. Fuhr *et al.* investigated how different brain regions synchronize their activity over space and time in autism, using resting-state brain scans from more than 1000 individuals. The autistic brain shows altered synchronization patterns in terms of variability, strength, and how synchronization spreads across the brain. Furthermore, these differences between autistic and typically developed individuals follow a hierarchical organization of the brain, running from sensory to higher cognitive regions.

Atypical neural connectivity has long been proposed to be a convergent neurobiological explanation behind the many “autisms” (1, 2, 3). Such atypical connectivity manifests in multiple ways ranging from genetic alterations to specific key synaptic proteins and convergent gene expression dysregulation affecting synaptic processes to observations of differences in macroscale structural and functional brain connectivity measured from neuroimaging data [e.g., (4, 5, 6, 7, 8, 9)]. Several types of intrinsic functional connectivity (FC) differences measured from resting-state functional magnetic resonance imaging (rs-fMRI) data have been observed throughout the literature. For example, based on static FC analysis (i.e., analysis of the entire rs-fMRI time series), autism can be characterized by alterations in whole-brain FC gradients (10), idiosyncratic connectivity profiles (11, 12, 13), mosaic patterns of hypo- and hyperconnectivity (7, 8, 9,14), and distance-dependent effects (2,15).

While static FC captures the average connectivity pattern over an entire scan, they assume that connectivity patterns are somewhat stationary over time. Alternatively, other studies have demonstrated that rs-fMRI showcases intrinsic nonstationarities or dynamics (16) that necessitate approaches that analyze the way dynamic FC patterns emerge and change over time (17). One such approach is metastability—a dynamic systems concept that captures the brain’s ability to flexibly switch between different functional configurations (18, 19, 20). Alterations in metastable dynamics have been used to distinguish between different psychiatric disorders, such as Alzheimer’s disease (21) and schizophrenia (22). In autism, metastability has been shown to offer a promising framework to link neuronal and behavioral flexibility. Core features such as restricted repetitive behavior and associated behavioral inflexibility have been linked to impaired neural state transitions (23), and recent evidence suggests that modifying this neuronal inflexibility can enhance behavioral flexibility (24).

However, descriptions of dynamic behaviors such as metastability are typically used to characterize the configuration of the whole brain. Concepts from other disciplines can extend metastability to capture spatial differences more effectively. One such framework is turbulence, an approach that draws on principles from fluid dynamics. Flow can be classified as either laminar or turbulent. Laminar flow is characterized by the smooth, orderly movement of fluid particles in parallel layers, resulting in predictable behavior. In contrast, turbulent flow is characterized by chaotic and irregular motion. The formation of vortices of different spatial scales makes turbulent flow highly unpredictable (25). An important property of turbulent flow is its ability to rapidly transfer energy across spatial scales, which allows information to propagate through the system more efficiently (26). Deco and Kringelbach (27) combined these properties with the theoretical framework of Kuramoto (28), who generalized turbulence beyond the limited domain of fluid dynamics to other physical systems including coupled oscillators. In their work on whole-brain modeling of brain dynamics, Deco *et al.* (29) have shown that one of the best models of the empirical data uses coupled Stuart-Landau oscillators. Their research models the brain as a system of coupled oscillators that can help identify turbulent markers in the brain with rs-fMRI. This demonstrated the value of the framework in providing a more comprehensive and fine-grained analysis of spatiotemporal synchronization dynamics. Specifically, the framework enables detailed examination of the variability and strength of functional synchronization, as well as the propagation cascades and spatial transfer/decay of synchronization. By integrating these complementary perspectives, the framework offers a more complete and nuanced characterization of functional synchronization dynamics.

In this work, we investigate functional synchronization dynamics across different spatial scales in rs-fMRI in autism using the turbulence-dynamics framework. We tested whether these dynamics differ between individuals with autism and those with typical development (TD) using rs-fMRI data from the ABIDE (Autism Brain Imaging Data Exchange) datasets.

## Methods and Materials

### Dataset

For the current study, we used data from the ABIDE I and II repositories (9,30). After quality control, correction for site effects and head motion, and the application of additional filtering criteria, the final sample consisted of 505 autistic individuals (mean age = 16.29 years, SD = 9.00) and 504 nonautistic individuals (mean age = 16.68 years, SD = 8.94) (Table S2 and Figure S2). Further details on quality control and filtering procedures are provided in the Supplemental Methods.

### fMRI Preprocessing

The preprocessing of the resting-state scans follows the pipeline described in Trakoshis *et al.* (31) and is outlined in the Supplemental Methods.

### Turbulence Analysis

Average blood oxygen level–dependent (BOLD) time-series data were extracted from a custom-made parcellation scheme, which combined the 7-network Schaefer-1000 atlas (32) and the Melbourne subcortical atlas (33). This custom-made atlas comprised 1054 regions, 54 of which were subcortical. After extraction of average BOLD time-series data for each parcel, data were then transformed to phase-space using the Hilbert transformation. Consequently, we obtained instantaneous phase information for each of the 1054 parcels as a function of time, which served as the basic input matrix for all subsequent analyses.

**Figure 1:**
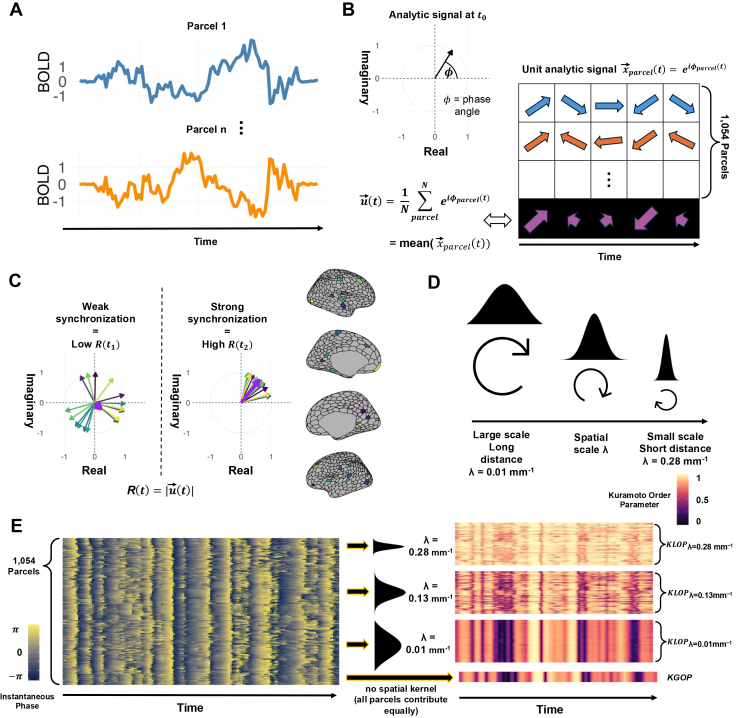
Methodology and turbulence dynamics framework. **(A)** The blood oxygen level–dependent (BOLD) time series for 1054 parcels was extracted using a combined 1000-parcel cortical atlas (34) and a 54-parcel subcortical atlas (35). **(B)** The BOLD time series for each parcel was transformed using the Hilbert transform to obtain the analytic signal. For a given parcel, each time point in the time series then contains 2-dimensional information that can be represented as a vector (top left panel). Each vector is composed of 2 parts: a real part (Re, x-axis), containing the value of the original BOLD time series, and an imaginary part (Im, y-axis), containing the 90° phase-shifted version of that value. Using Euler’s formula, this vector is expressed in terms of instantaneous phase (angle ϕ) and amplitude. Because our focus is on synchronization, we extracted only the phase information by normalizing each vector to unit length, resulting in the complex unit analytic signal *x*(*t*) (right panel). At each time point, the unit analytical signal for each parcel (illustratively shown for 2 parcels in blue and orange) was summed and then divided by the total number of parcels to obtain the mean unit vector *u*(*t*) (purple arrows at the bottom). **(C)** The Kuramoto order parameter *R* is defined as the magnitude (or length) of *u*(*t*) and varies as a function of time. The 2 panels show a graphical representation of *R* at 2 different time points, *t*_1_ and *t*_2_. Each colored vector resulting from the Hilbert-transformed time series represents the unit analytic signal *x*(*t*) of a parcel of the Schaefer-Tian parcellation. The location of each parcel in the brain can be seen in the plots on the right. The bold purple vector represents the mean unit vector; its length is *R*. When the colored vectors are dispersed, they cancel each other out. The resulting sum is relatively small, leading to a small *R* (left-hand panel, weak synchronization). When the vectors are aligned in phase, the resulting summed vector is relatively long. This leads to a higher *R* (right-hand panel, strong synchronization). **(D)** Spatial scales were introduced to investigate synchronization and information transmission across different distances in the brain. Low values of λ correspond to larger spatial scales/longer distances in the brain. Conversely, as λ increases, the spatial scale decreases, and synchronization at more local levels is investigated. **(E)** To compute a localized version of the Kuramoto order parameter for a single parcel *n*_0_, the instantaneous phase of all other parcels (left panel) is combined with a spatial kernel function (middle panel) that weighs their contributions based on their Euclidean distance to *n*_0_. For each parcel *n* and each kernel width λ, the weighted unit analytic signal is summed, following the standard Kuramoto formula **(B)**. The magnitude of the resulting complex average yields the Kuramoto local order parameter (KLOP), which varies as a function of time. The right-most panel shows 2-dimensional heatmaps of the KLOP for 3 different values of λ. Each heatmap shows how the KLOP changes over time, with the rows representing different parcels of the brain and the columns representing successive time points. Higher KLOP values, indicating stronger synchronization, are shown in brighter colors, while lower values (weaker synchronization) appear darker. The λ decreases (i.e., the spatial scale increases) from the top-most to the bottom-most heatmap. This results in a gradual loss of temporal and spatial detail. It reflects a shift from a more focal kernel capturing local synchronization (top heatmap) to a broader kernel assessing more global synchronization (bottom heatmaps). To contrast the Kuramoto global order parameter (KGOP) with the KLOP, the KGOP is visualized as a 1-dimensional vector at the very bottom. The KGOP is computed without any weighting kernel and therefore captures the phase synchronization of the whole brain, where each parcel contributes equally to the magnitude of synchronization. Note that even though some spatial detail is preserved, the KLOP approximates the KGOP for each parcel on very large spatial scales (i.e., λ = 0.01 mm^−1^).

**Figure 2:**
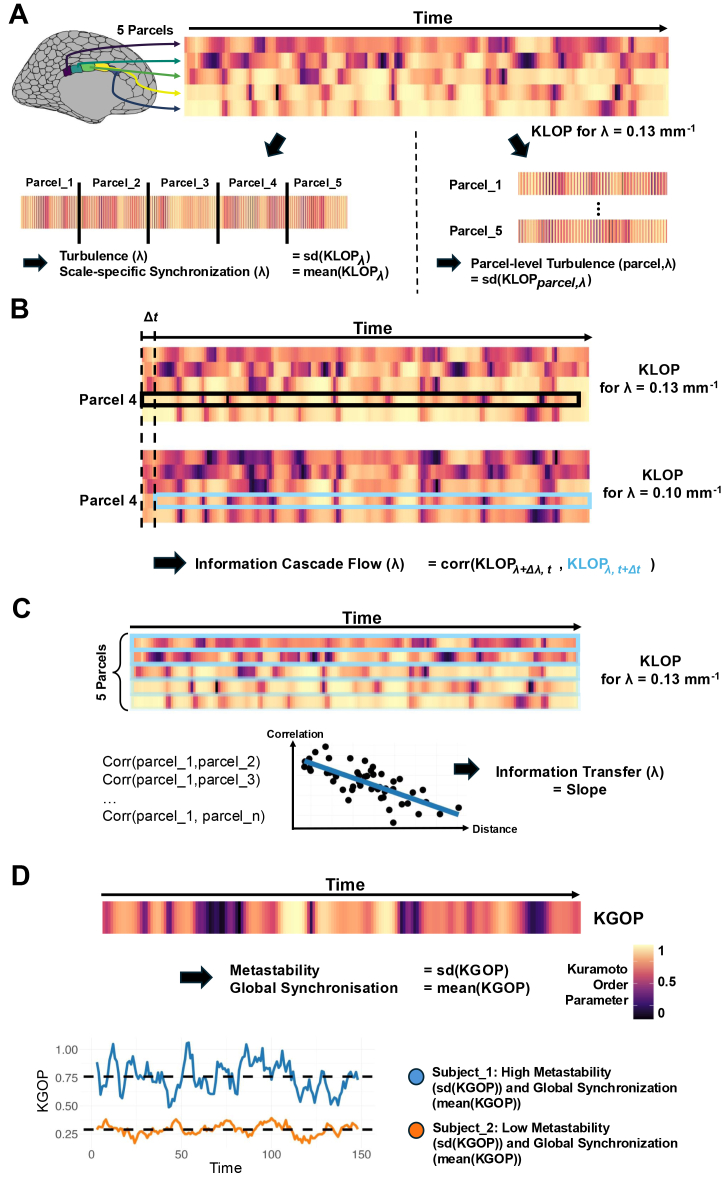
Description of higher-order and whole-brain summary statistics. **(A)** illustrates the computation of turbulence, parcel-level turbulence, and scale-specific synchronization statistics. The heatmap at the top shows the evolution of the Kuramoto local order parameter (KLOP) for a given spatial scale (λ = 0.13 mm^−1^) for 5 representative parcels over time. Each row represents a parcel, with the distance between parcels increasing from top to bottom. Each column represents a time point, with the color scale representing KLOP values. To compute the scale-specific synchronization and turbulence for a given spatial scale, this 2-dimensional matrix is vectorized (bottom left of the panel). The standard deviation of the resulting 1-dimensional vector over time is the turbulence for this spatial scale, while the mean is the scale-specific synchronization. The bottom right of the panel shows how parcel-level turbulence is computed for each parcel and spatial scale. The standard deviation of a parcel’s KLOP for a given λ is that parcel’s turbulence. Panel **(B)** visualizes the computation of the information cascade flow. The 2 heatmaps show the evolution of the KLOP for 2 spatial scales (λ = 0.13 mm^−1^ and λ = 0.10 mm^−1^) for 5 representative parcels over time. The KLOP for parcel 4 is highlighted with a box. The information cascade flow is calculated by advancing the KLOP values of parcel’s 4 higher spatial scale (λ = 0.10 mm^−1^, blue box, bottom heatmap) by 1 timestep to the right and correlating the resulting vector with the parcel’s 4 original KLOP vector at the adjacent lower spatial scale (λ = 0.13 mm^−1^, black box, top heatmap). This correlation value is computed for each parcel and then averaged to obtain the information cascade flow for this λ pair. Panel **(C)** visualizes the computation of the information transfer statistics. For a given spatial scale (e.g., λ = 0.13 mm^−1^), the correlation between each parcel and all other parcels is computed. The resulting correlation values are fitted with a line as a function of distance. The slope of this linear fit then represents the information transfer value for this spatial scale. Panel **(D)** shows the computation of whole-brain summary statistics for metastability and global synchronization. The 1-dimensional vector is the Kuramoto global order parameter (KGOP). Metastability statistics are the standard deviation of the KGOP over time, and global synchronization statistics are the mean of the KGOP over time. Metastability and global synchronization for 2 hypothetical subjects are visualized at the very bottom.

This phase-resolved representation of whole-brain activity across all 1054 parcels served as the basis for applying the turbulence dynamics framework. This framework has recently been used to characterize dynamic synchronization patterns in human brain activity (27). Figure 1 illustrates the framework’s methodology. In brief, the framework quantifies synchronization variability, strength, and propagation across spatial scales (λ), ranging from ∼3.6 mm (1/0.28 mm^−^^1^) to ∼100 mm (1/0.01 mm^−^^1^), in 10 steps. For each parcel, time point, and spatial scale, we computed the Kuramoto local order parameter (KLOP), yielding a 3-dimensional matrix whose dimensions correspond to parcels, time points, and lambda (λ) values (see Figure 1A–E). These matrices capture the degree of local synchronization between a parcel and its λ-defined neighborhood. Based on these matrices, we derived several higher-order statistics (see Figure 2 and Video S1 for the temporal evolution of the KLOP). Table 1 summarizes all the derived measures and their conceptual rationale. See the Supplemental Methods for detailed explanations of each measure. The computational procedures for extracting these statistics follow those of Escrichs *et al.* (34) and Martínez-Molina *et al.* (35), while the original whole-brain turbulence framework is detailed in Deco and Kringelbach (27). All analyses were implemented in MATLAB (version R2024b; The MathWorks, Inc.).

**Table 1:** Summary Table of All Metrics

Statistic	Spatial Scale	Interpretation
Global Synchronization	Brainwide/global	Overall strength of synchronization across the whole brain
Scale-Specific Synchronization	Scale-specific (local to global)	Strength of synchronization at different spatial scales
Metastability	Brainwide/global	Temporal variability of synchronization across the whole brain
Turbulence	Scale-specific (local to global)	Temporal variability of synchronization at different spatial scales
Parcel-Level Turbulence	Parcel-level (with spatially varying neighborhood influence)	Temporal variability of synchronization within individual parcels at different spatial scales
Information Transfer	Scale-specific (local to global)	Rate at which synchronization decays across space
Information Cascade Flow	Scale-specific (local to global)	Efficiency of synchronization propagation from lower to higher spatial scales

### Statistical Analysis

Group differences and autism-specific associations in whole-brain summary measures (metastability and global synchronization) were assessed using linear models controlling for age, sex, mean framewise displacement (FD), and diagnosis or Autism Diagnostic Observation Schedule (ADOS) score, including interaction terms with age. ADOS scores were normalized across modules by converting them to percentage-of-maximum scores, thereby enabling direct comparability across participants (see the Supplemental Methods for details). For measures specific for spatial scales (turbulence, scale-specific synchronization, information cascade flow, and information transfer), we used linear mixed-effects models with λ modeled as a repeated-measures factor with random intercepts and slopes. Post hoc tests were false discovery rate (FDR) corrected (see Tables S5 and S6 for model specifications). Parcel-level group differences in turbulence were evaluated by computing Cohen’s *d* across all 1054 parcels after regressing out age, sex, and mean FD. Parcels with the largest differences were assigned to canonical resting-state networks (RSNs) to identify network-level enrichment. To complement these discrete network analyses, we also quantified the similarity between parcel-level effect-size maps and continuous gradients of cortical organization, including the sensorimotor-association (S-A) and FC gradients (36,37). Gradient correspondence was assessed using rank-based correlations and spatial permutation (spin) tests to account for spatial autocorrelation (38,39), with FDR correction applied across λ levels. For details on the statistical analysis, see the Supplemental Methods.

## Results

### Autism Is Characterized by Higher Variability in Synchronization at Local/Small Spatial Scales

**Figure 3:**
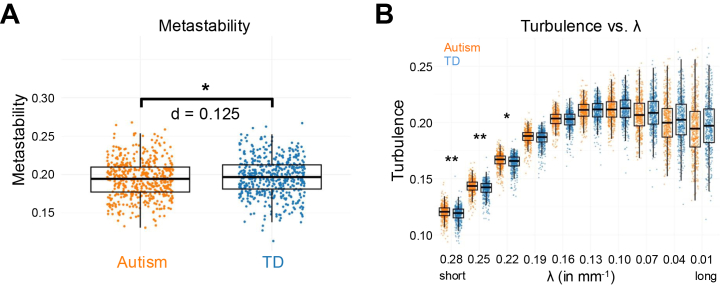
Increased synchronization variability at small spatial scales in autism. Panel **(A)** shows a significant effect of a group difference between autism and typical development (TD) in whole-brain variability of synchronization (e.g., metastability). The autism group shows a small effect of lower levels of metastability at the level of the whole brain compared with the TD group. **(B)** Turbulence levels of autism (orange) vs. TD (blue) as a function of spatial scale (λ). Turbulence levels are significantly increased in the autism group compared with the TD group for smaller spatial scales (i.e., high λ = shorter distances). As the spatial scale increases (i.e., larger distances), the pattern of group difference on turbulence shows a tendency to reverse. Error bars represent 95% CIs. Asterisks denote level of statistical significance after false discovery rate multiple comparison correction (∗*p* < .05, ∗∗*p* < .01).

In this study, we applied the turbulence-dynamics framework to rs-fMRI data from the ABIDE dataset (autism *n* = 505; TD *n* = 504). First, we examined differences in synchronization variability using a metric called metastability [i.e., sd(KGOP)]. Metastability differed significantly between groups (Figure 3A and Table S4) (*F*_1,1003_ = 3.92, *p* = .048, *d* = 0.13), with lower values in the autism group, indicating reduced temporal variability in global synchronization. In the autism group (*n* = 465), ADOS percentage scores showed no association with metastability (Figure S3A and Table S4). Because metastability reflects synchronization at the broadest spatial scale (i.e., the whole brain), in our next analysis, we used a distance-specific weighting parameter, denoted as λ, to assess group differences in functional synchronization variability [i.e., sd(KLOP) or turbulence] on different spatial scales (λ). Here, smaller λ values index larger spatial scales, whereas larger λ values reflect smaller, more localized scales. Although there was no main effect of diagnosis (*F*_1,1041_ = 0.09, *p* = .763), we observed a significant λ × diagnosis interaction (*F*_1,1198_ = 8.58, *p* = .003) (Figure 3B and Table S5). Post hoc comparisons showed that the autism group exhibited greater synchronization variability at small, localized spatial scales. However, as the spatial distance increased, temporal fluctuations in synchronization normalized and were not different in the TD and autism groups. This result of higher variability in functional synchronization at smaller or more localized spatial levels in autism is striking given that at the whole-brain level (Figure 3A), the difference is reversed in autism (e.g., less variability in functional synchronization in autism). Analyses within the autism group revealed an analogous profile, i.e., no main effect of ADOS scores on turbulence levels but a significant λ × ADOS interaction (Figure S3B–D; Table S5). Individuals with higher autistic trait severity showed increased turbulence at smaller spatial scales, with this association disappearing at larger scales. Together, these findings demonstrate a marked scale-specific divergence in synchronization dynamics in autism—heightened turbulence locally and reduced variability globally—with stronger effects in individuals with greater autistic trait severity.

### Variability of Parcel-Level Functional Synchronization Changes as a Function of Well-Known Large-Scale Functional Networks in Autism

**Figure 4:**
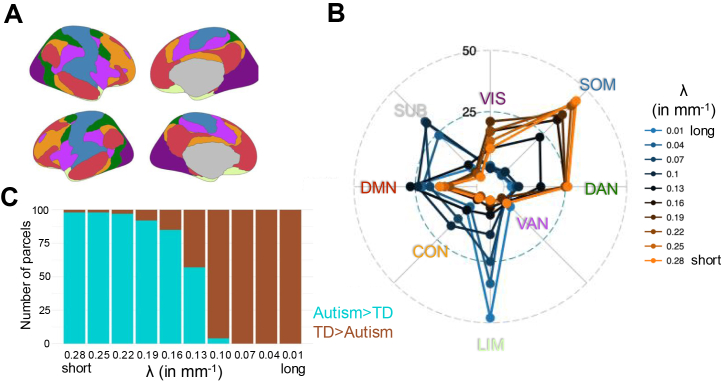
Parcel-level turbulence effects across well-known resting-state networks (RSNs). Panel **(A)** shows brain surface plots of 7 Yeo networks (39) and 1 subcortical network (35) utilized for analysis. Panel **(B)** shows spider plots that describe how the top 100 parcels with the largest group differences at a given spatial scale λ were distributed across RSNs. The different spider plots represent results at each spatial scale denoted by λ. Values within the plots indicate the percentage of parcels of the top 100 that fall within a specific network. Panel **(C)** is a bar plot showing the percentage of parcels of the top 100 with a typical development (TD) > autism (brown) or autism > TD (cyan) directionality of group difference. CON, control network; DAN, dorsal attention network; DMN, default mode network; LIM, limbic network; SOM, somatomotor network; SUB, subcortical areas; VAN, ventral attention network; VIS, visual network.

To better understand how variability in functional synchronization manifests within large-scale functional networks in autism, we examined parcel-level turbulence. Parcel-level turbulence quantifies the temporal variability of synchronization for each parcel at a given spatial scale λ. For each λ, we identified the 100 parcels showing the largest absolute group differences in turbulence and assigned them to 1 of 7 RSNs defined by Yeo *et al.* (40) or to a subcortical network (33) (Figure 4A). At the smallest spatial scales (i.e., larger *λ* values), parcels showing greater turbulence in autism were predominantly located in sensorimotor and attention networks (Figure 4B and Table S6). As the spatial scale increased (i.e., λ decreased), the pattern shifted; parcels showing reduced turbulence in autism were increasingly concentrated in subcortical, default mode, limbic, and control networks. This scale-dependent distribution mirrors the overall group-level turbulence findings (Figures 3B and 4C). Specifically, at the shortest distances (e.g., λ = 0.28 mm^−^^1^), participants in the autism group exhibited higher turbulence than participants in the TD group, whereas at larger distances (e.g., λ = 0.01 mm^−^^1^), the effect reversed, with the TD group showing greater turbulence. This pattern was consistent across different parcel counts (*n* = 50, 100, or 200) (Figure S4).

Together, these findings indicate that autism is characterized by elevated turbulence in localized dynamics within sensorimotor and attention networks but reduced turbulence at broader spatial scales within subcortical, limbic, and higher-order association networks such as the default mode and control systems.

### Variability of Parcel-Level Functional Synchronization Maps Onto S-A and Functional Gradients in Autism

**Figure 5:**
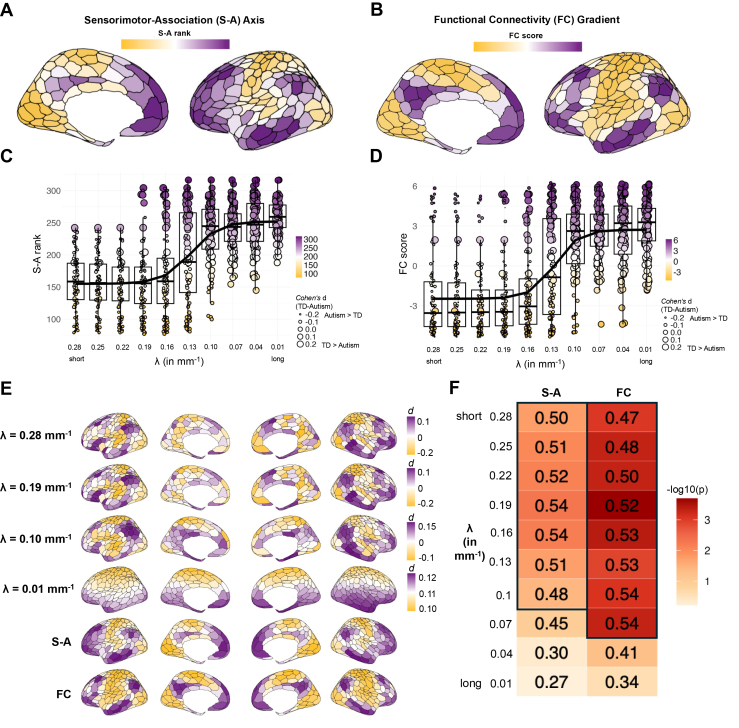
Similarity between synchronization variability group differences and S-A or FC gradient organization. Panels **(A)** and **(B)** show the S-A axis **(A)** and the FC gradient **(B)** maps. Panels **(C)** and **(D)** show ranks (shown via the y-axis and coloring of the dots) for the top 100 parcels (dots) over each spatial scale λ (x-axis). The size of the dots represents the standardized effect size for a group difference, with the smallest dots representing an autism > typical development (TD) difference, while positive-signed effect sizes (i.e., TD > autism) are denoted with larger dots. The predicted values for each λ in the fitted logistic model are plotted as the smooth black curve. **(E)** Similarities of Cohen’s *d* of parcel-level turbulence and S-A and functional gradient maps. Parcel-level turbulence maps are displayed for 4 spatial scales, with the top panel displaying the smallest spatial scale. Golden colors indicate increased synchronization variability in the autism group compared with the TD group. Conversely, purple colors indicate decreased synchronization variability. **(F)** This heatmap shows the correlation values (Spearman’s ρ) of parcel-level turbulence and S-A and functional gradient maps for each spatial scale. The colors in the heatmap are −log10(*p*); red indicates lower *p* values, and orange indicates higher *p* values. For each spatial scale, the Spearman rho value is written inside the cell. Spatial scales for which there was a statistically significant correlation with S-A or FC gradients are outlined in black.

The previous analysis investigated the effects of synchronization variability over well-known RSNs. RSNs represent discrete network divisions and only give one angle for examining intrinsic functional brain organization. It is known that the brain exhibits a hierarchical gradient-like organization across many modalities. One such gradient is the S-A axis (36) (Figure 5A), a neurodevelopmentally significant organizational axis implicated in hierarchical cortical maturation (36,41) and autism (42) potentially reflecting downstream consequences of early genomic patterning differences (6,43). The S-A axis aligns closely with primary FC gradients (37) (Figure 5B). Examining both gradients provides a complementary perspective on how turbulence-related group differences are embedded within cortical hierarchy beyond RSN-based classifications.

We first investigated where the top 100 parcels showing the largest absolute turbulence group differences were positioned along the S-A and FC gradients across spatial scales λ. Because λ indexes spatial distance (larger λ = more localized scales; smaller λ = broader scales), we assessed whether the gradient location of these parcels systematically varied with λ. To quantify this, we modeled the relationship between parcel gradient rank and λ using linear, polynomial, and logistic models to capture potential nonlinearities, including S-shaped trajectories. Across both gradients, the top 100 parcels showed systematic shifts in gradient position as a function of spatial scale (Figure 5C, D and Table S7). These shifts were best captured by an S curve; at small spatial scales (higher λ), parcels were concentrated at the sensorimotor end of the S-A and FC axes, whereas at larger spatial scales (lower λ), the dominant parcels shifted toward association cortex. The direction of group differences also varied by scale, as indicated by effect size sign. At small spatial scales, effect sizes were predominantly negative, reflecting higher turbulence in autism. At larger spatial scales, effect sizes reversed, with the TD group showing higher turbulence than the autism group. Thus, the regions contributing most strongly to turbulence group differences occupy different positions along the cortical hierarchy depending on λ, sensorimotor regions at small scales and association regions at larger scales.

To complement the top parcel analysis, we also examined similarity between full parcel-level turbulence group difference maps and S-A or FC gradients without selecting parcels. Using Spearman correlations and spatial permutation (spin) tests, we found significant correspondence between turbulence maps and both gradients across most spatial scales, except at the largest scales (λ = 0.07 − 0.01 mm^−^^1^) (Figure 5F and Table S8). One possible reason for the lack of similarity at large spatial scales could be that such finer-grained spatial patterning breaks down when spatial scales become too large. This can be seen in the effect size maps at λ = 0.01 mm^−1^ in Figure 5E because the spatial effects here are too coarse and resemble simpler dorsal-ventral gradient patterning rather than spatially more complex and fine-grained patterning consistent with S-A and FC gradients. However, overall, these findings provide further evidence that differences in synchronization variability align with well-established S-A and FC gradients across the whole cortex. Autism can be characterized by higher synchronization variability in sensorimotor areas and lower synchronization variability in association areas.

### Weaker, Less Spatially Correlated, and Rapidly Decaying Functional Synchronization Over Space in Autism

**Figure 6:**
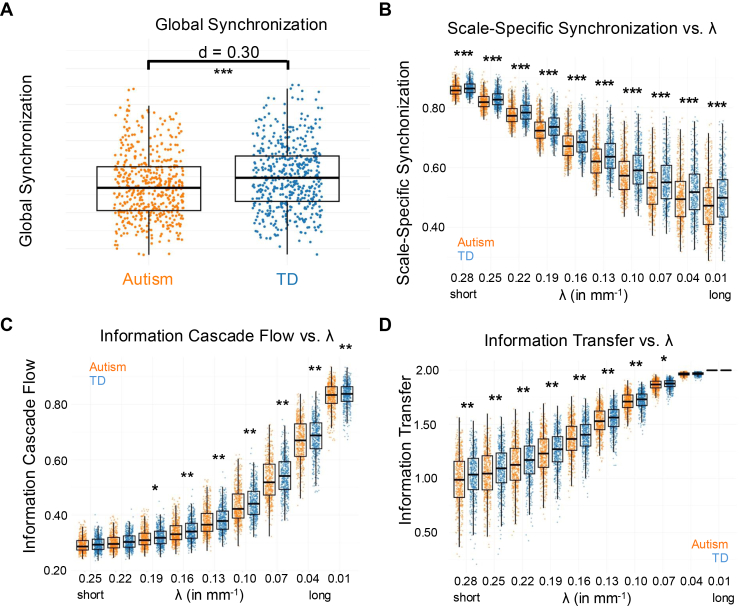
Group differences in synchronization strength, information cascade flow, and information transfer. Panel **(A)** shows a reduction in whole-brain global synchronization strength in autistic individuals (orange) compared with individuals with typical development (TD) (blue). Panel **(B)** shows that reduced synchronization strength in autism is also apparent at each spatial scale depicted by λ. **(C,D)** Information cascade flow **(C)** and information transfer levels **(D)** are decreased in autistic individuals compared with TD individuals for most spatial scales (λ shown on the x-axis). Differences in information cascade flow levels are most pronounced on longer distances, while differences in information transfer levels are most pronounced on smaller distances. Asterisks denote level of statistical significance after false discovery rate (FDR) multiple comparison correction (∗*p* < .05, ∗∗*p* < .01, ∗∗∗*p* < .001).

In addition to assessing synchronization variability as a function of spatial scale, our framework allowed us to evaluate synchronization strength. First, we assessed synchronization strength at the whole-brain level [i.e., mean(KGOP) or global synchronization]. We found a highly significant difference between groups (*F*_1,1003_ = 22.62, *p* = .000002, *d* = 0.300) (Figure 6A and Table S4), with weaker whole-brain synchronization in the autism group compared with the TD group. We also investigated the strength of functional synchronization over time as a function of spatial scale (λ)—what we call scale-specific synchronization [i.e., mean(KLOP)]. Here we found a main effect of diagnosis (*F*_1,996_ = 20.90, *p* = .000005) (Figure 6B and Table S5) as well as a significant λ × diagnosis interaction (*F*_1,1007_ = 15.23, *p* = .0001). Post hoc tests revealed that scale-specific synchronization levels were significantly lower in autistic individuals than in TD individuals for all spatial scales, with the difference being most pronounced over longer distances. We also found a significant age × diagnosis interaction (*F*_1,1003_ = 10.05, *p* = .002) on scale-specific synchronization strength. While in the TD group, synchronization strength increased with age, in the autism group, it stayed constant (Figure S5). Furthermore, our analysis within the autism group revealed a negative association of ADOS scores with global and scale-specific synchronization levels (Figure S6A, B; Tables S4 and S5). These results indicate that the strength of synchronization was weaker in the autism group than in the TD group for all distances and that individuals with higher ADOS severity scores exhibited weaker synchronization.

In addition to examining synchronization strength and variability, we assessed how synchronization propagates across spatial scales. To test this, we used a measure called information cascade flow, quantifying the correlation between synchronization at adjacent spatial scales (e.g., corr[KLOP_λ1_, KLOP_λ2_]). We observed a significant main effect of diagnosis (*F*_1,1020_ = 9.04, *p* = .003) but no λ × diagnosis interaction (*F*_1.2532_ = 2.12, *p* = .15). Post hoc tests indicated consistently lower information cascade flow in autism across nearly all spatial scales, with the largest differences at broader scales (lower λ) (Figure 6C and Table S5). In the autism group, ADOS scores were not associated with information cascade flow (Figure S6C; Table S5). These findings suggest that long-range propagation of synchronization is reduced in autism.

Finally, we examined the rate of decay in functional synchronization over spatial scales, indicated by the measure we call information transfer (e.g., linear modeling of KLOP correlations over parcels as a function of distance). Here, we found a main effect of diagnosis (*F*_1,995_ = 9.07, *p* = .003) and a significant λ × diagnosis interaction (*F*_1,1073_ = 10.31, *p* = .001). Follow-up tests showed reduced information transfer in autism at all but the largest spatial scales (λ = 0.01 mm^−^^1^, λ = 0.04 mm^−^^1^) (Figure 6D and Table S5). In the autism group, ADOS percentage scores were negatively associated with information transfer, and this relationship varied across λ (Figure S6D; Table S5). Thus, synchronization decayed more rapidly over short and intermediate distances in autism, especially among individuals with higher autistic trait severity. Taken together, these results reveal 3 alterations in autism: 1) reduced synchronization strength across all spatial scales, 2) weakened propagation of synchronization across medium and large distances, and 3) faster spatial decay of synchronization at shorter distances. Moreover, both weaker synchronization strength and accelerated decay are amplified with increasing symptom severity as measured by ADOS scores.

## Discussion

In this study, we applied the turbulence-dynamics framework to examine functional synchronization patterns on different spatial scales in rs-fMRI scans of the brains of autistic individuals. Our findings reveal robust evidence of altered functional synchronization patterns within and between spatial scales. Compared with typically developing individuals, we observed increased variability (i.e., increased turbulence), faster spatial decay (i.e., lower information transfer), and decreased functional synchronization strength over short distances in autism. This increased variability is primarily attributed to the sensorimotor and unimodal areas of the brain. At long distances, the strength of functional synchronization remains reduced. However, we also found that the influence of lower spatial scales on adjacent, higher spatial scales of functional synchronization was reduced (i.e., information cascade flow). Furthermore, the marginally decreased variability of synchronization at long distances in autistic individuals is primarily found in the association and transmodal areas of the brain.

How do these observed results from the turbulence framework fit with longstanding ideas about altered FC as a convergent phenomenon explaining the neurobiology behind the autisms? One theme in the literature suggests that autism can be characterized by local overconnectivity versus long-range underconnectivity (1,2,44). For example, using a static FC approach that measures connectivity over the entire time series, Weber *et al.* (15) found evidence supporting this view on local overconnectivity and long-range underconnectivity. However, a meta-analysis by Hull *et al.* (44) suggests that evidence supporting this theme is mixed and may be due to methodological differences. As an alternative to static connectivity approaches, in this work, we used a dynamic approach that not only enables characterization of synchronization strength but also allows for statements about variability in synchronization. Regarding the strength of synchronization, we found evidence that partially supports the view of underconnectivity. However, our observations show that underconnectivity is present at both local and long-range distances. The framework can also be used to make observations about the variability in connectivity over time. Here, we found that variability in connectivity depends on distance; shorter distances show increased variability, while at longer distances, group differences in variability become much smaller and may change in directionality. Thus, the turbulence framework allows for a more complete description of connectivity in the autistic brain not only in terms of strength over an assumed period but also about how variable connection strength is in autism. Future work using this framework in autism could help confirm whether such observations are a hallmark of autism or perhaps a subset of autistic cases with specific biological or phenotypic profiles.

### From Local Hypervariability to Global Decoupling: Multiscale Connectivity Alterations in Autism

The combination of local increases in synchronization variability, faster dissipation of synchronization over space, and reduction in synchronization strength in autism may signal something important about the underlying neurobiology residing locally at the level of synapses. Past work has shown that normative processes controlled by mTOR (mechanistic target of rapamycin)-dependent signaling induce synaptic spine pruning in early brain development (43). These processes are likely altered in autism (45,46) and could cause observed phenomenon in postmortem data such as increased synaptic spine density in autism (47). These and other microstructural phenomena, such as increased cell density (48, 49, 50), could help to explain the increased synchronization variability (i.e., higher turbulence), faster dissipation of synchronization over space (i.e., information transfer), and reduction in the strength of local functional synchronization.

In addition to these effects on the local level, other findings from this study such as reduced information cascade flow may also hint at the idea of global decoupling in autism. Information cascade flow can be interpreted as the way synchronization travels from smaller to larger spatial scales. A reduction in this measure indicates that information at local levels does not cascade well across longer distances. This result could implicate issues with longer-range anatomical connections such as white matter tracts. Compatible with this idea, diffusion imaging studies have found reduced levels of integrity of long-range white matter tracts (i.e., reduced fractional anisotropy and diffusivity) in autism at ages such as those in the current work (51, 52, 53). Thus, abnormalities in long-range white matter pathways may constrain synchronization propagation, particularly at larger spatial scales. These ideas also match well with insights from computational models. For example, Deco *et al.* (54) demonstrated that long-range connections increase cascade levels in the brain. Therefore, disruption of these connections in autism may explain the reduced capacity to integrate synchronization across different spatial scales (i.e., reduced information cascade flow levels).

Taken together, these results suggest that in the brains of autistic individuals, local overconnectivity may give rise to excessively turbulent synchronization dynamics at smaller spatial scales. This locally unstable pattern seems unable to sustain coherence over longer distances, resulting in reduced synchronization strength and variability. At larger scales, long-range underconnectivity and alterations in white matter anatomy may further exacerbate these disruptions, yielding a decoupled and overly rigid brain organization at longer distances. This scale-dependent dichotomy is significant because it extends previous reports of scale-independent hypervariability in functional synchronization (55).

### Altered Synchronization Patterns Across Varying Distances Are Related to Hierarchical Brain Organization

Moving beyond distance-dependent connectivity patterns, a substantial body of research suggests that the human brain is hierarchically organized along a gradient that spans sensorimotor to association regions (i.e., the S-A axis) (36,37). The S-A axis has been identified as atypical in autism, as indicated by a contracted functional hierarchy observed in rs-fMRI data (10). We found additional evidence implicating the S-A axis in autism with the turbulence framework applied to rs-fMRI data. We observed increased short-range functional variability in sensorimotor areas alongside decreased long-range functional variability in association areas. These results may match well with observations that suggest that a diverse range of phenomena across the information-processing hierarchy are different in autism (42). The S-A axis is also strongly linked to cortical maturation trajectories. Markers such as the age at which peak myelination growth occurs (56) and parvalbumin expression patterns (57) vary systematically along this axis (36). Thus, the differences in synchronization variability that we observed may reflect differences in the timing of cortical development along this axis. Early-developing sensorimotor regions may undergo refinement that promotes heightened local coupling, while later-developing association regions may exhibit reduced large-scale synchronization. Therefore, the turbulence alterations that we observe may index developmental differences in the way functional organization unfolds along the S-A axis.

### Limitations and Future Directions

There are some caveats and limitations relevant to this work that could be addressed in future studies. First, although we observed robust evidence for case-control differences, most of these differences were small in effect size. Therefore, we also extended our analysis to investigate relations within the autism group. The results suggest that analyzing synchronization dynamics on different spatial scales is informative because most measures varied significantly with ADOS scores. However, because effects are also rather small in the autism group, it seems that variance in these metrics may not be captured best by spectrum-like dimensional variation. While small effect sizes comparing group differences are consistent with the findings of many other studies (58), a more fruitful approach is to see whether turbulence metrics can be used to delineate subtypes (58, 59, 60, 61, 62). Second, we opted for maximal statistical power in the current study, utilizing all available data from the ABIDE datasets. Future work on independent datasets with similarly large sample sizes would be useful to test the replicability of such effects. Third, most of the results of this study are based on the association between turbulence metrics and either group differences, ADOS scores, or hierarchical gradients. To illuminate potential causal mechanisms, future work modeling the sources of differences between the 2 groups is needed.

Fourth, because this work relies on the ABIDE datasets, it is limited to observations in older autistic individuals, many of whom have average-to-high intellectual ability. Studies in younger cohorts and across a broader range of symptom severity and intellectual functioning will be necessary to generalize these findings to the wider autistic population. A further open question concerns whether the developmental trajectories of turbulence dynamics differ between autistic and typically developing individuals. Prior work on developmental changes in FC has already pointed to age-related effects; within-network hyperconnectivity observed in younger autistic individuals tends to diminish or even reverse into hypoconnectivity with age (63). Moreover, typical age-related increases in segregation and decreases in integration (64) appear inverted in autism (65). Our results on synchronization strength are partially consistent with these age-related patterns and underscore the importance of considering developmental factors. From adolescence into adulthood, typically developing individuals show increasing connectivity strength, whereas in autism, synchronization strength remains comparatively stable. However, these observations are based on age effects across the entire cohort rather than within individuals. Therefore, longitudinal studies will be essential to determine how these dynamics evolve over time in each group.

### Conclusions

Our findings demonstrate that functional synchronization profiles in autism depend on spatial scale and vary systematically along the S-A gradient. Increased local turbulent dynamics fail to propagate a stable synchronization pattern across spatial scales. As a result, autistic individuals show a decoupled and overly rigid synchronization profile over longer cortical distances. When projected onto the S-A axis, these effects manifest as increased synchronization variability within sensorimotor regions but reduced variability within association cortices. As next steps, more research is needed to replicate these findings and to further explore differences in the turbulence framework outcomes across clinically relevant autism subtypes.

## Supplementary Material

Tables S1–S8

Key Resources Table
