# Supplementary material for: Dynamic Functional Synchronization Profiles in Autism Differ by Spatial Scale and Along Hierarchical Cortical Gradients
Source: Biol Psychiatry Glob Open Sci. 2026 Jul 8;6(5):100787. doi: 10.1016/j.bpsgos.2026.100787 (PMC13475223; doi:10.1016/j.bpsgos.2026.100787)
Supplement: Supplementary file 1 [file mmc1.pdf]

SUPPLEMENTARY INFORMATION

Dynamic Functional Synchronization Profiles in Autism Differ by Spatial Scale and Along Hierarchical Cortical Gradients

Fuhr *et al.*

Supplementary Information

Supplementary Methods

Dataset

We analyzed resting-state fMRI data from the ABIDE dataset (1,2). From the total ABIDE I and II datasets, we included data from 24 sites. Data from the University of Michigan was excluded from the analysis due to its imbalanced distribution of Autism and TD (~6:1 Autism to TD).

The ABIDE dataset varies in terms of scanner types and other imaging parameters (see details of imaging parameters by site in Supplementary Table S1). Since these types of variation between imaging sites can affect BOLD data and connectivity measurements, we implemented a site correction technique (*ComBat,* 3). The ComBat model included variables such as diagnosis, sex, age, mean FD and a diagnosis×age interaction as covariates in the model. To examine the effect of ComBat for removing variance due to site, we compared the first principal component from a PCA analysis before and after implementing ComBat. PC1 before ComBat implementation showed substantial variability between sites ($F(23,985) = 11.99$, $p < 0.001$) (Supplementary Figure S1a). This variability was eliminated after ComBat implementation, demonstrating the effectiveness of ComBat at removing site variation ($F(23,985) = 1.053$, $p = 0.394$) (Supplementary Figure S1b). Moreover, we conducted Fisher z-tests on the group differences in the correlation between mean FD and each measure, to confirm that movement in the scanner did not affect the two groups differently. The results showed that mean FD had a significant effect on each measure, and that this effect did not differ between the groups (Supplementary Table S3).

We implemented further quality checks and filtering criteria after preprocessing of the resting-state scans. Subjects who demonstrated poor image quality following visual inspection (e.g., failed spatial normalization or co-registration) were excluded from the study. Furthermore, subjects with a mean framewise displacement (FD) above 0.5mm were excluded from the study. To minimize confounds of motion on our analysis, we undertook a further investigation of group differences of mean FD between autism and TD. A statistically significant increase in movement was observed in the Autism group in comparison to the TD group ($t(1157) = 4.17$, $p = 0.00003$, $d = 0.26$). To address this issue, a matching process was performed on both groups according to their FD values. This was achieved by employing the *matchit* function from R, with a caliper of 0.05 (4). Following the implementation of this matching process, the differences observed between the groups in terms of FD were found to be non-significant ($t(1010) = 0.6165$, $p = 0.538$, $d = 0.039$). After applying these filtering criteria, n = 1009 subjects remained. 505 of these subjects were autistic individuals (mean age = 16.29 years, age SD = 9.00) and 504 were non-autistic individuals (mean age = 16.68 years, age SD = 8.94) (Supplementary Table S2; Supplementary Figure S2).

Imaging parameters and fMRI preprocessing

Preprocessing of the resting-state data was split into two components: core preprocessing and denoising. Core preprocessing was implemented with AFNI (5) using the tool speedypp.py (6). This core preprocessing pipeline included the following steps: (i) slice

acquisition correction using heptic (7th order) Lagrange polynomial interpolation; (ii) rigid-body head movement correction to the first frame of data, using quintic (5th order) polynomial interpolation to estimate the realignment parameters (three displacements and three rotations); (iii) obliquity transform to the structural image; (iv) affine co-registration to the skull-stripped structural image using a gray matter mask; (v) nonlinear warping to MNI space (MNI152 template) with AFNI 3dQwarp; (vi) spatial smoothing (6 mm FWHM); and (vii) a within-run intensity normalization to a whole-brain median of 1000. Core preprocessing was followed by denoising steps to further remove motion-related and other artifacts. Denoising steps included: (viii) wavelet time series despiking ('wavelet denoising'); (ix) confound signal regression including the six motion parameters estimated in (ii) and their first order temporal derivatives. Regression of motion was achieved using AFNI 3dBandpass with the –ort argument. The wavelet denoising method has been shown to mitigate substantial spatial and temporal heterogeneity in motion-related artifacts that manifest linearly or nonlinearly and can do so without the need for data scrubbing (7). Data scrubbing (i.e. volume censoring) cannot be used in our time-series-based analyses here as such a procedure breaks up the temporal structure of the time-series. Wavelet denoising is implemented with the Brain Wavelet toolbox. After preprocessing, the time-series data were detrended and mean-centred. A second-order Butterworth bandpass filter was then applied to pass data within the 0.008–0.08 Hz frequency band.

Turbulence dynamics framework

To formalize the turbulent dynamics framework, we adopt the approach of viewing the brain as a complex system of coupled oscillators (8). In this model, the time-series of the 1,054 brain parcels serve as fundamental elements of the system. Information for each parcel is provided by the instantaneous phase of the Hilbert-transformed time series (Figure 1a-b). To quantify the magnitude of synchronization at a given timepoint, we computed the Kuramoto order parameter, denoted by the letter R. This order parameter was computed either uniformly over the whole brain (Kuramoto *global* order parameter, 'KGOP'), or over a range of spatial scales (denoted by the coefficient λ), which resulted in the Kuramoto *local* order parameter ('KLOP') (Figure 1c–e). KGOP values indicate the extent to which the entire brain is in phase synchrony at a given time. In contrast, KLOP values indicate the degree to which a given parcel is in phase synchrony with a selected portion of its neighboring parcels at a given time. The 'selection' of these neighboring parcels is determined by the spatial scale λ, which ultimately acts as a kernel function for the given brain parcel. λ is defined at ten values ranging from 0.01 to 0.28 mm^{-1}, at intervals of 0.03 mm^{-1}. λ indicates the decay rate in the exponential kernel in the definition of the Kuramoto order parameter (with units of mm^{-1}). Considering the characteristic decay length as the distance at which the kernel has decayed to $1/e \approx 37\%$ of its original value, λ ranges from ~3.6mm ($1/0.28mm^{-1}$) to ~100mm ($1/0.01mm^{-1}$) distance in the brain. Conceptually, λ indicates the extent to which neighboring parcels contribute to the level of synchronization (Figure 1d). A higher value of λ (e.g., 0.28 mm^{-1}) corresponds to a more constricted weighing kernel, where influence diminishes more rapidly with increasing distance. Consequently, parcels further away contribute less to the KLOP of the specified brain parcel. Conversely, a lower value of λ (e.g., 0.01 mm^{-1}) corresponds to a wider weighing kernel. Therefore, the influence diminishes more gradually with increasing distance, and parcels that are further away contribute more to the KLOP of the specified brain area (Figure 1e; Supplementary Movie S1).

First, we computed four higher-order statistics using the *Kuramoto Local Order Parameter* (KLOP) for each λ, making them susceptible to spatial scales:

(1) Turbulence captures the spatiotemporal extension of metastability. For each spatial scale, a slice of the three-dimensional KLOP matrix is vectorized, and the standard deviation is computed on the resulting vector. This is therefore a measure of the variability of synchronization on a given spatial scale (Figure 2a).

(2) Scale-specific synchronization captures the strength of synchronization at a given distance. For each spatial scale, a slice of the three-dimensional KLOP matrix is vectorized, and the mean is computed on the resulting vector. This is therefore a measure of the central tendency of synchronization at a given spatial scale (Figure 2a).

(3) Information cascade flow captures the propagation of synchronization from a lower spatial scale to its immediately adjacent higher spatial scale. This is calculated by advancing the KLOP values of a given parcel's higher spatial scale by one timestep (lower λ-value) and correlating the resulting vector with the parcel's original KLOP vector at the adjacent lower spatial scale (higher λ-value). This correlation value is computed for each parcel and then averaged. It measures the extent to which synchronization at the lower spatial scale influences, or is reflected in, synchronization at the higher spatial scale for each pair of adjacent spatial scales (Figure 2b).

(4) Information transfer captures the spatial extent over which synchronization is maintained within the brain at a given spatial scale. It is quantified by correlating the KLOP values across all pairs of parcels within that scale. These correlation values are then modeled as a function of inter-parcel distance, using a linear fit. The slope of this fit reflects the rate at which synchronization strength decays with distance (Figure 2c).

Second, we computed two whole-brain summary statistics using the *Kuramoto Global Order Parameter* (KGOP). The KGOP is computed using the same rationale as the KLOP. Unlike the KLOP, however, the KGOP captures the synchronization of the whole brain at a given moment in time, instead of the synchronization for each individual parcel. Consequently, it does not use a weighting kernel in its computations, so all parcels have the same weight. The KGOP is then computed as the magnitude of the mean unit vector u(t) (Figure 1b-c). Note, however, that for the lowest λ values (e.g., $\lambda = 0.01\ mm^{-1}$, indicating very large spatial kernels), the KLOP approaches the KGOP since the kernel function becomes wider and all parcels therefore contribute almost equally to the Kuramoto order parameter (note the similarity between the KLOP ($\lambda = 0.01\ mm^{-1}$) and KGOP heatmaps at the bottom of Figure 1e). We computed two summary-statistics:

(5) Metastability is a heuristic measure of the configuration of the attractor landscape underlying complex brain dynamics and captures the temporal variation in whole-brain synchronization. Here, it is computed as the standard deviation of the KGOP over time (Figure 2d).

(6) Global synchronization captures the central tendency of the whole-brain synchronization and is computed as the mean of the KGOP over time (Figure 2d).

Third, parcel-level turbulence was computed for different values of λ. For each λ, parcel-level turbulence is calculated using the standard deviation of a parcel's KLOP vector over time. This captures how a parcel’s synchronization varies over time at different spatial scales.

Statistical Analysis

Before conducting any statistical analyses, we transformed the ADOS scores available in ABIDE into ADOS percentage scores. To do so, we divided each subject's ADOS score by the maximum possible ADOS score for that subject, depending on the ADOS module used (acting as an upper ceiling). This number was then multiplied by 100 to obtain a percentage representing the ADOS score achieved by each subject. This made it possible to compare subjects who took tests from different modules with each other.

To assess group differences and differences within the autism group in whole-brain summary measures (i.e., metastability and global synchronization), we used linear models implemented with the *lm* function in R. For the analysis of group differences, the model included sex, mean FD, diagnosis, age, and the diagnosis×age interaction as independent variables. For the analysis within the autism group, the model included sex, mean FD, ADOS, age, and the ADOS×age interaction as independent variables. The dependent variable was one of metastability or global synchronization. To assess group differences and differences within the autism group over measures using different spatial scales (i.e., turbulence, scale-specific synchronization, information cascade flow and information transfer) we used linear mixed effect models, implemented with the *lmer* function in the *lme4* R library. For the analysis of group differences, fixed effects included diagnosis, λ, age, sex, and mean FD, along with interaction terms for diagnosis×λ and diagnosis×age. Random effects were modeled for the repeated measure of λ with random intercepts and slopes. Post-hoc pairwise comparisons were used to compare groups at each value of λ by computing the estimated marginal mean from the model and performing pairwise *t*-tests for each spatial scale. For the analysis restricted to the autism group, fixed effects included ADOS, λ, age, sex, and mean FD, along with interaction terms for ADOS×λ and ADOS×age. Random effects were modeled for the repeated measure of λ with random intercepts and slopes. Post-hoc inference was performed by estimating the marginal ADOS trend at each λ and testing whether these slopes differed from zero using z-tests. To account for multiple comparisons, p-values from the post-hoc tests were corrected using the False Discovery Rate (FDR) method.

To assess differences in parcel-level turbulence between TD and autism across λ levels, we first regressed out all the variance related to our covariates mean FD, sex and age. Based on the resulting residuals, we then calculated Cohen's d as the difference between the two groups for each of the 1,054 parcels. We then extracted the 100 parcels that showed the largest absolute value difference in parcel-level turbulence for each λ. For each spatial scale, these 100 parcels were attributed to one of seven RSNs (9) and a subcortical network to determine which networks are enriched for the largest differences in parcel-level turbulence.

Discrete RSNs demonstrate that the brain is intrinsically functionally organized. However, to provide a different, yet complementary perspective to RSNs, we also examine continuous gradients as another key principle for explaining how the brain is hierarchically organized. Thus, in addition to looking at turbulence group differences relative to known RSNs, we also examined how similar turbulence group differences would be with continuous gradients of hierarchical cortical organization, such as the sensorimotor-association (S A) axis (10). The S-A axis is a multimodal hierarchical gradient that describes how many features (e.g., macrostructural, microstructural, functional, metabolic, transcriptomic, and evolutionary) vary across the cortex (10). As an example, it is well known that the S-A axis is quite similar in topography to the primary functional connectivity (FC) gradients measured with rsfMRI (11). The S-A axis is also neurodevelopmentally sensitive (10,12) and thus allows an interesting

perspective on neurodevelopmental disorders like autism. One pole of the S-A or FC axes comprises areas involved in sensorimotor and unimodal processing, while the other pole consists of association areas and transmodal processing areas. The S-A axis is hypothesized to be a neurodevelopmentally important axis for hierarchical brain organization in autism (13) and may be one of the downstream convergent consequences of early genomic cortical patterning differences in autism (14,15). In these analyses, we evaluate the similarity between parcel-level turbulence group difference maps and S-A and FC gradient maps. To achieve this, we first identified the top 100 parcels with the strongest group differences in parcel-level turbulence. Ranking parcels to get a top 100 involved first computing a rank score for all 1,000 cortical parcels from the Schaefer parcellation. We then computed a weighted sum of the Sydnor gradient rank score from the five nearest neighbors for each of the 1,000 parcels, with the weight dependent on the distance. Then, we selected the top 100 parcels showing largest differences in effect sizes for each λ and assigned a gradient score to each of the 100 parcels. We then fitted linear, polynomial, and logistic models to best characterize how the gradient score of the top 100 parcels (i.e., the dependent variable) could be predicted from λ values (i.e. the independent variable). We then performed model selection between these three models and utilized a combination of the residual sum of squares (RSS), Akaike information criterion (AIC) and Bayesian information criterion (BIC) criteria to identify the best fitting model. Once the best model has been identified, we then report inferential statistics to describe how the gradient may relate to effect sizes over spatial scales. In addition to examining the top 100 parcels, we also adopted a different approach of simply testing for correlations between parcel-level turbulence group difference maps and S-A and FC maps. For this analysis, we used the same Schaefer 400 parcellation as Sydnor et al. (10) and computed parcel-level turbulence values for each of the 400 parcels by taking the weighted average of the five closest parcels from the initial parcellation of 1,000 cortical parcels. Standardized effect size (Cohen's d) was then computed for every parcel, and this was repeated at each level of λ. Statistical comparisons were then made between parcel-level Cohen's d turbulence maps and the S-A or FC maps. These were implemented by computing Spearman ρ and then evaluating statistical significance via permutation p-values from 'spin tests' (10,000 repetitions) that preserve spatial autocorrelation (16,17). To account for multiple comparisons, we corrected the p-values from the permutation tests using the false discovery rate (FDR) method.

Supplementary Figures

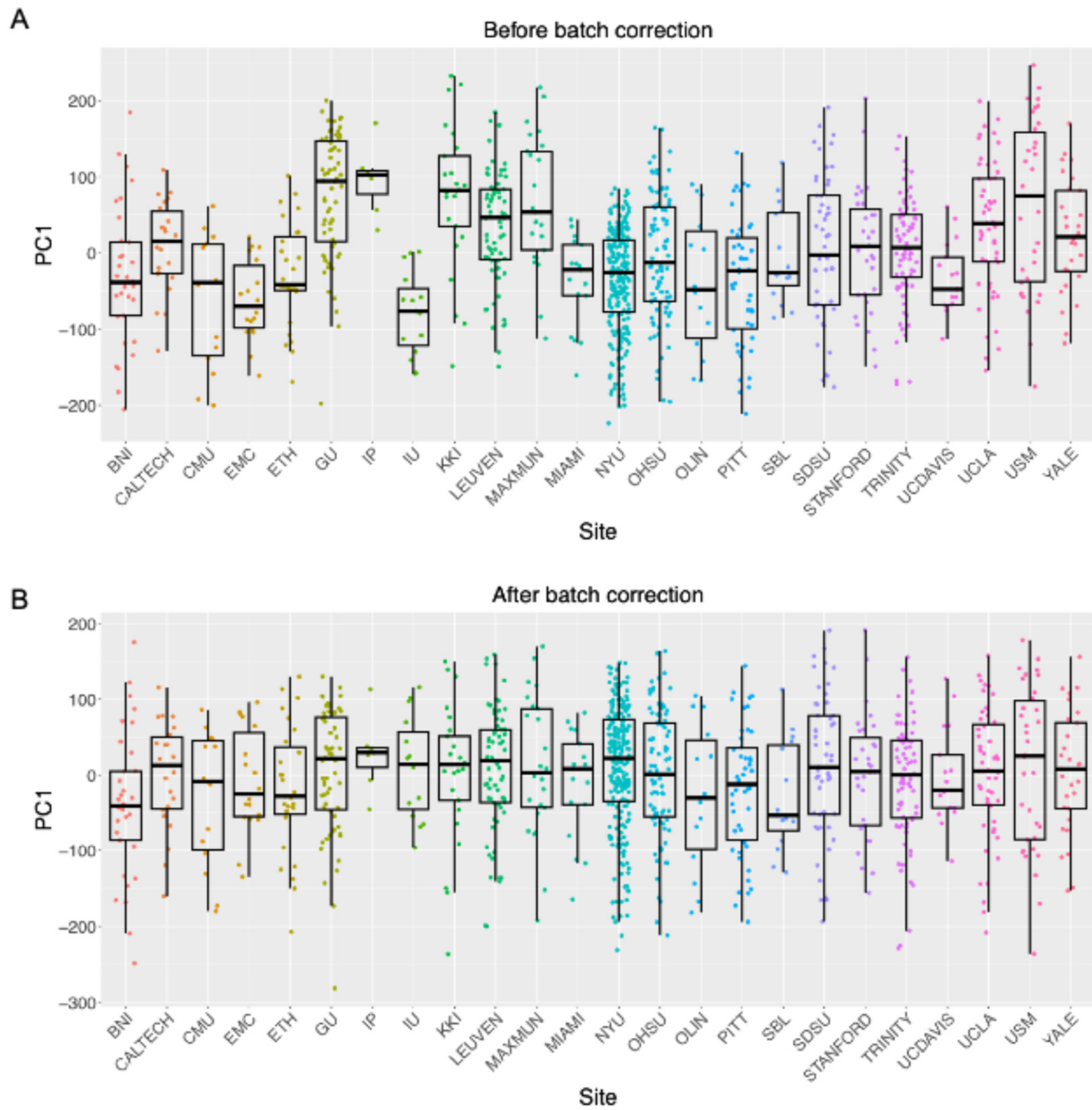

Figure S1. *Boxplots of the first principal component values of all combined information processing measures for each site before (A) and after (B) batch correction.*

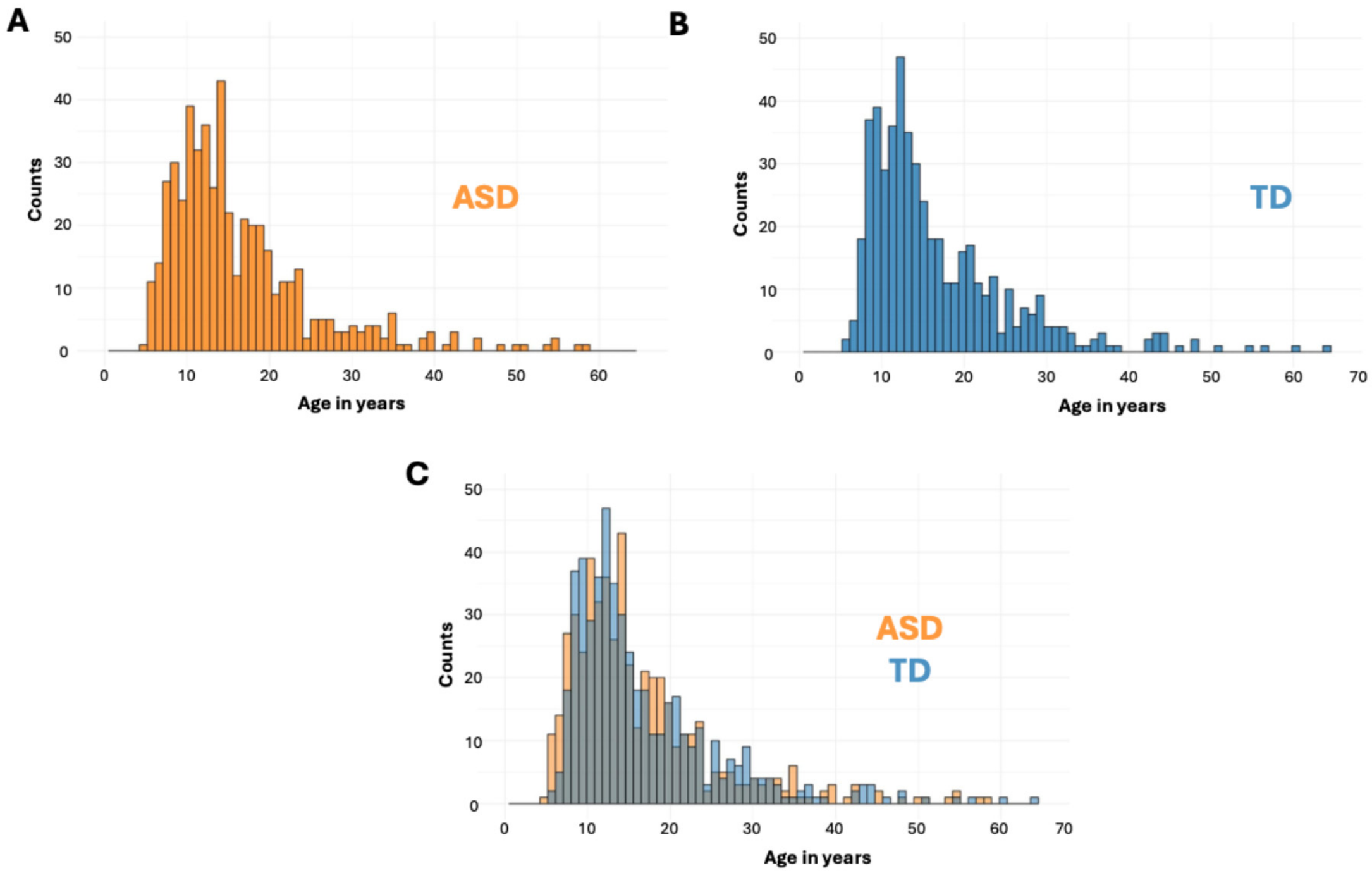

Figure S2. *Histograms of age (in years) for the ASD group (A), TD group (B), and both groups overlaid (C).*

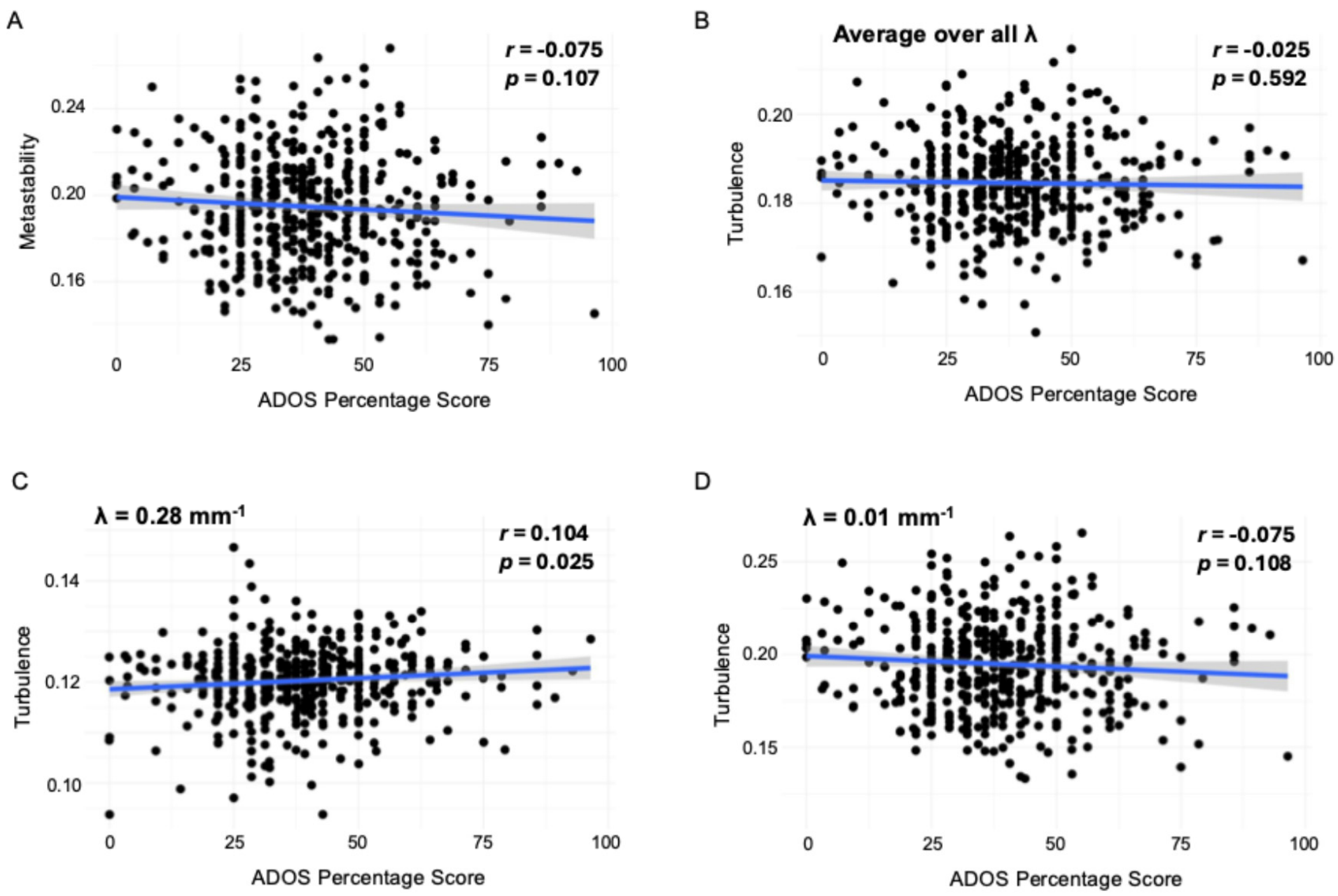

Figure S3. *Associations between ADOS percentage scores and metastability (A), turbulence averaged over all λ (B), turbulence for the smallest spatial scale (λ = 0.28* mm^{-1}*) (C) and turbulence for the largest spatial scale (λ = 0.01* mm^{-1}*) (D). The solid blue line shows the linear regression of each measure on ADOS severity. The shaded band indicates the 95% confidence interval of the estimated mean. Variability of synchronization over the whole brain (A), averaged over spatial scales (B) and larger spatial scales (e.g., λ = 0.01* mm^{-1}*) (D) showed no significant correlation with ADOS scores. Variability of synchronization showed a positive correlation with ADOS scores on the smallest spatial scales (e.g., λ = 0.28* mm^{-1}*) (C). See also Supplementary Table S6 for statistics.*

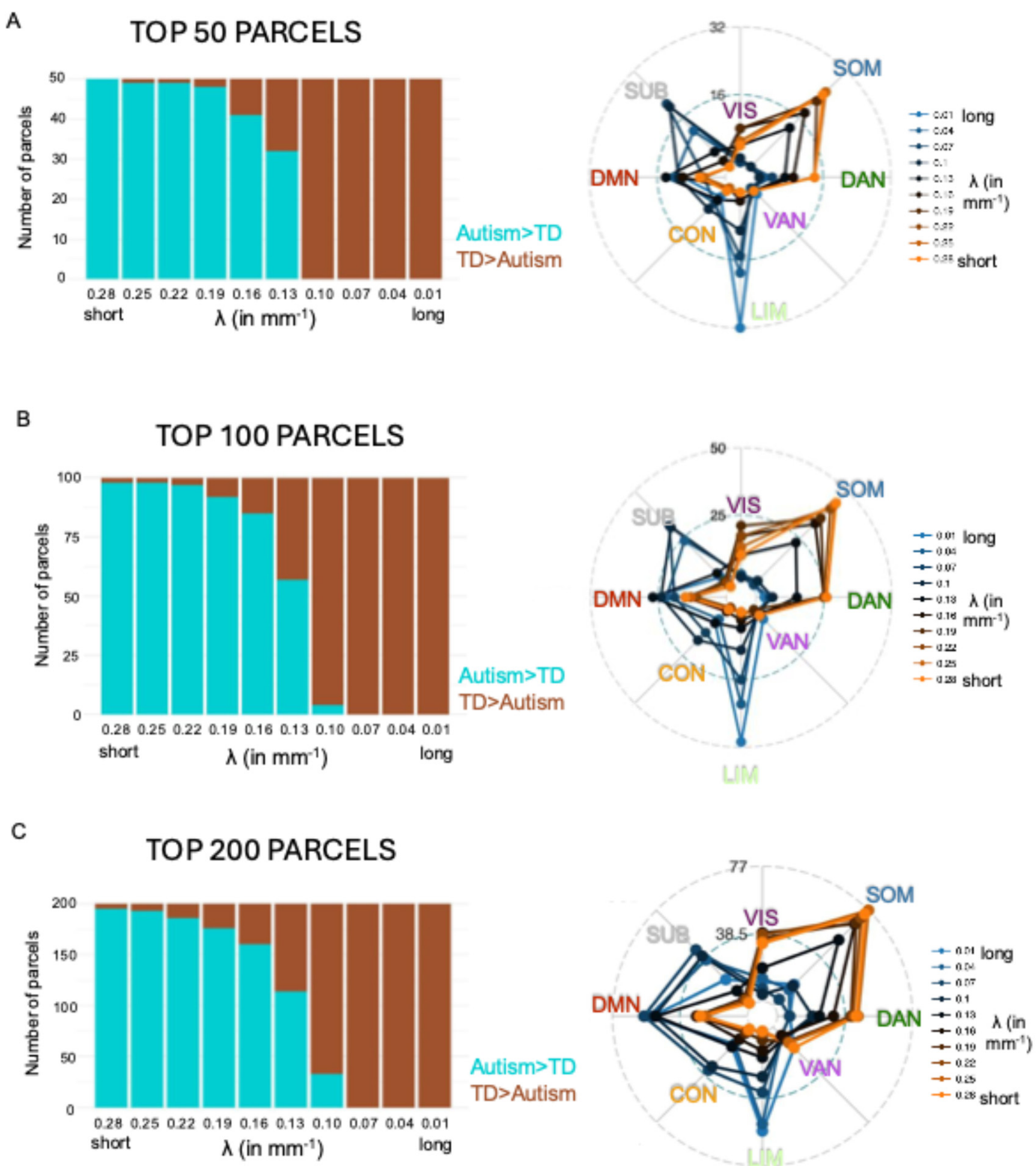

Figure S4. Parcel-level turbulence effects for different numbers of parcels. *Parcel-level turbulence analyses reveal similar results when choosing 50 (panel A), 100 (panel B and main text) or 200 parcels (panel C). The spider plots describe how the top-n parcels with largest group differences at a given spatial scale λ were distributed across RSN networks. The different spider plots represent results at each spatial scale denoted by λ. Values within the plots indicate the percentage of parcels out of the top-n that fall within a specific network. The bar plots show the percentage of parcels out of the top-n parcels with a TD>Autism (brown) or Autism>TD (cyan) directionality of group difference. Abbreviations: VIS, visual network; SOM, somatomotor network; DAN, dorsal-attention network; VAN, ventral-attention network; LIM, limbic network; CON, control network; DMN, default-mode network; SUB, subcortical areas.*

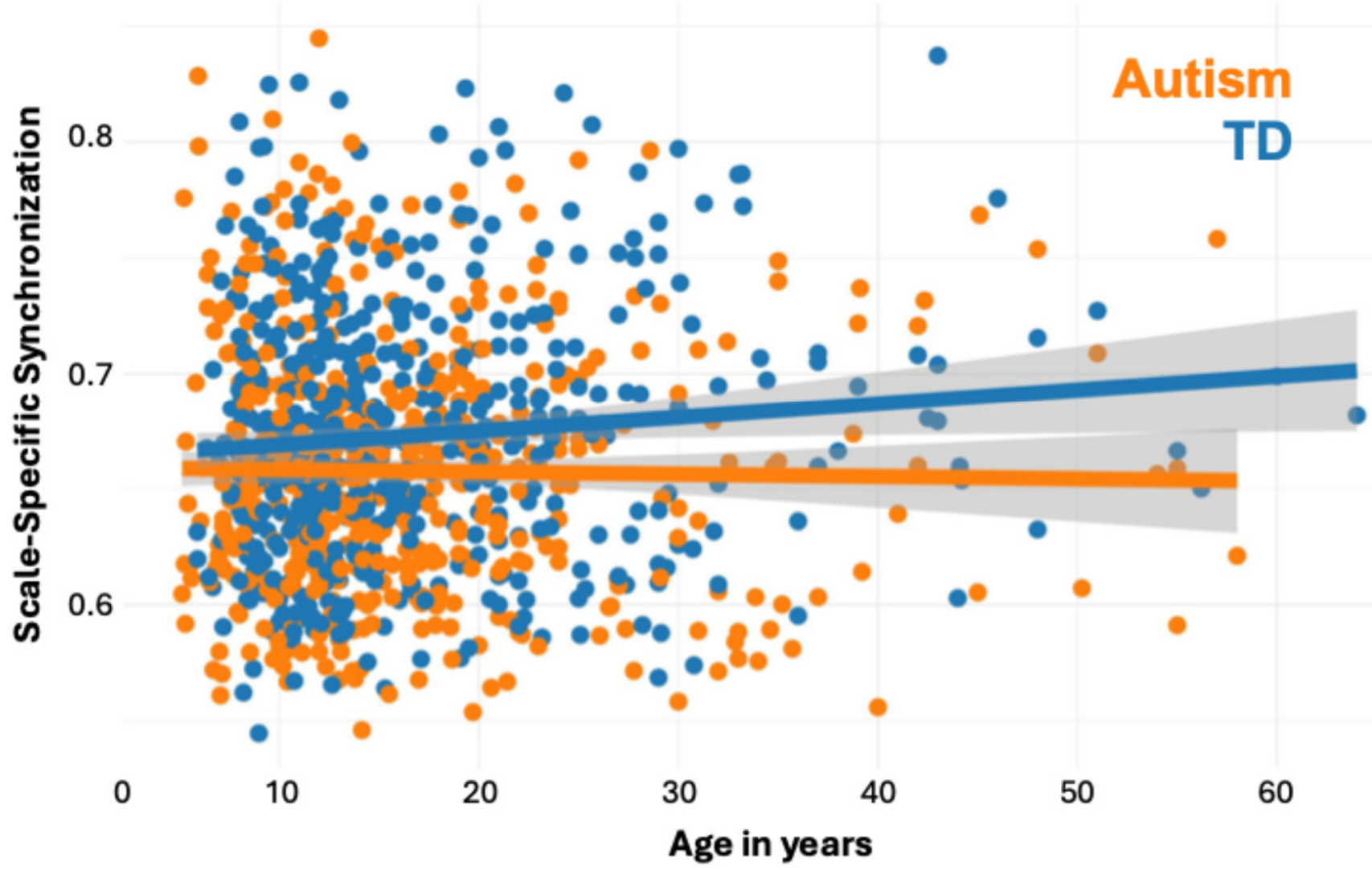

Figure S5. *Associations between scale-specific synchronization and age (in years) for both the ASD group (orange) and the TD group (blue). The solid lines show the linear regression of scale-specific synchronization against age, and the shaded band indicates the 95% confidence interval of the estimated mean for each group. The values for scale-specific synchronization were averaged across all spatial scales, to obtain a clear visual of the diagnosis×age interaction effect (Supplementary Table S6).*

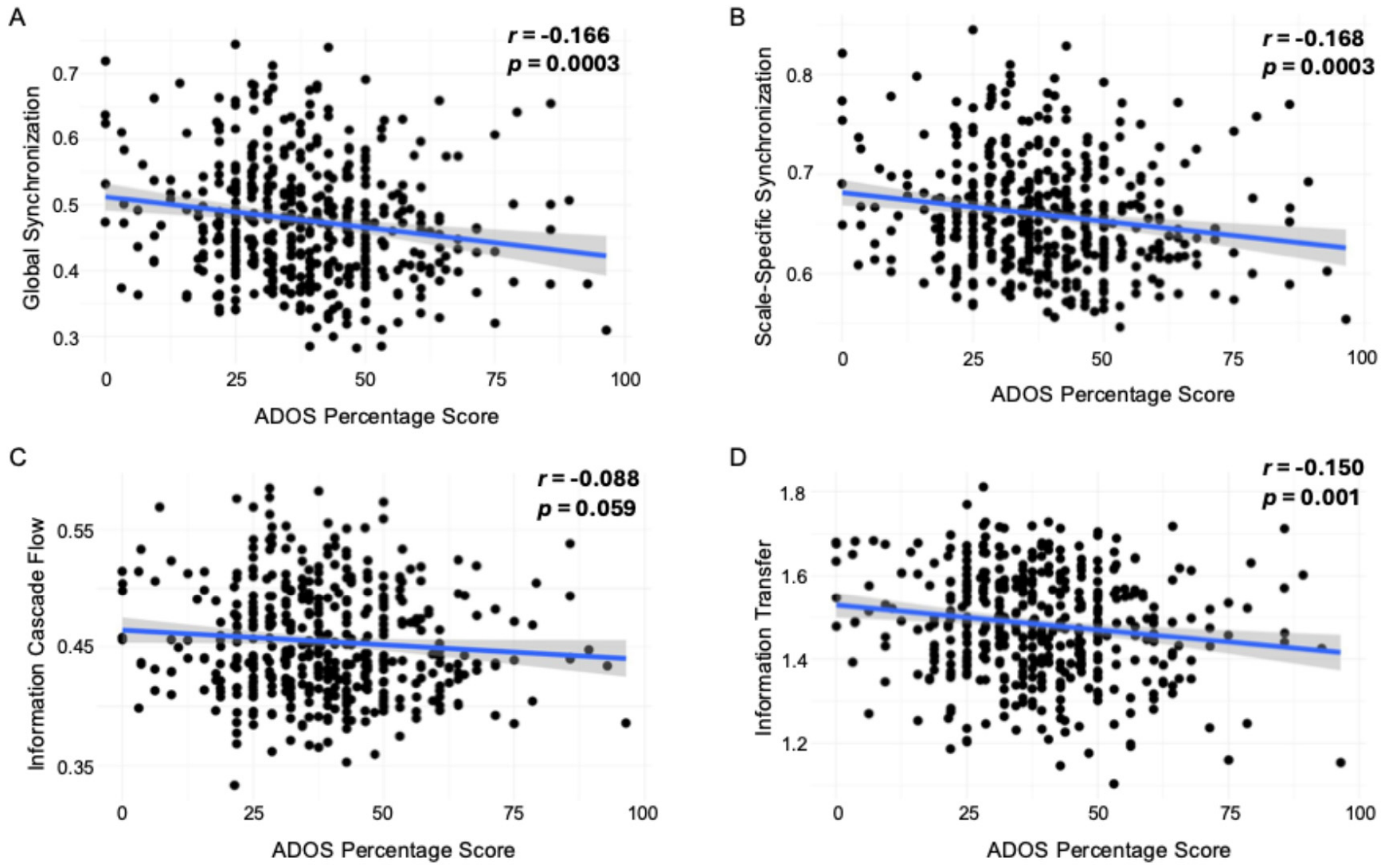

Figure S6. *Associations between ADOS percentage scores and global synchronization (A), scale-specific synchronization (B), information cascade flow (C) and information transfer (D). The solid blue line shows the linear regression of each measure against ADOS severity, and the shaded band indicates the 95% confidence interval of the estimated mean. For plots B, C and D, the values for each measure were averaged across all spatial scales. All measures showed significant negative correlations with ADOS severity scores, while for information cascade flow this was only marginally significant (Supplementary Tables S5, S6).*

Supplementary Table Legends

Supplementary Table 1: *MRI acquisition parameters for different sites in the ABIDE dataset.*

Supplementary Table 2: *Sample size and descriptive statistics for the ABIDE dataset. Age is stated in years.*

Supplementary Table 3: *Inferential statistics for Fisher z-tests between r-values of correlations between mean FD and each measure.*

Supplementary Table 4: *Inferential statistics for global synchronization and metastability.*

Supplementary Table 5: *Inferential statistics for scale-specific synchronization, turbulence, information cascade flow, and information transfer measures.*

Supplementary Table 6: *Parcel-level turbulence differences measured in effect sizes (Cohen's d)*

Supplementary Table 7: *Model comparisons for S-A and functional connectivity gradient, along with best model (logistic) statistics.*

Supplementary Table 8: *Correlation between parcel-level turbulence difference maps (TD-autism) and S-A or FC gradients (Schaefer400 parcellation).*

Supplementary Movie legend

Supplementary Movie 1: *This movie shows the spatiotemporal evolution of the phases and the Kuramoto Local Order Parameter (KLOP) for each of the 1,054 parcels. In the middle panel, each dot represents a single parcel. The y-value of each parcel corresponds to its phase value at a given timepoint. The brain rendering in the corner of the movie shows how this phase-information translates into the KLOP for each parcel at a given timepoint. Brighter colors represent high synchronization (high KLOP) for a given parcel, whereas darker colors represent low synchronization (low KLOP). The panels in the top row show a focal spatial kernel (*$\lambda = 0.28$ mm^{-1}*), producing a finer, more mosaic-like spatiotemporal evolution of the KLOP. Conversely, the bottom panels show a broader spatial kernel (*$\lambda = 0.01$ mm^{-1}*), displaying a nearly brain-wide spatiotemporal evolution of the KLOP.*

References

1. Di Martino A, Yan CG, Li Q, Denio E, Castellanos FX, Alaerts K, et al. The autism brain imaging data exchange: towards a large-scale evaluation of the intrinsic brain architecture in autism. Mol Psychiatry. 2014 Jun;19(6):659–67. doi:10.1038/mp.2013.78
2. Di Martino A, O'Connor D, Chen B, Alaerts K, Anderson JS, Assaf M, et al. Enhancing studies of the connectome in autism using the autism brain imaging data exchange II. Sci Data. 2017 Mar 14;4:170010. doi:10.1038/sdata.2017.10 PubMed PMID: 28291247; PubMed Central PMCID: PMC5349246.
3. Leek JT, Johnson WE, Parker HS, Jaffe AE, Storey JD. The sva package for removing batch effects and other unwanted variation in high-throughput experiments. Bioinformatics. 2012 Mar 15;28(6):882–3. doi:10.1093/bioinformatics/bts034 PubMed PMID: 22257669; PubMed Central PMCID: PMC3307112.
4. Ho DE, Imai K, King G, Stuart EA. **MatchIt** : Nonparametric Preprocessing for Parametric Causal Inference. J Stat Soft. 2011;42(8). doi:10.18637/jss.v042.i08
5. Cox RW. AFNI: Software for Analysis and Visualization of Functional Magnetic Resonance Neuroimages. Computers and Biomedical Research. 1996 Jun;29(3):162–73. doi:10.1006/cbmr.1996.0014
6. Kundu P, Inati SJ, Evans JW, Luh WM, Bandettini PA. Differentiating BOLD and non-BOLD signals in fMRI time series using multi-echo EPI. NeuroImage. 2012 Apr 15;60(3):1759–70. doi:10.1016/j.neuroimage.2011.12.028
7. Patel AX, Kundu P, Rubinov M, Jones PS, Vértes PE, Ersche KD, et al. A wavelet method for modeling and despiking motion artifacts from resting-state fMRI time series. NeuroImage. 2014 Jul 15;95:287–304. doi:10.1016/j.neuroimage.2014.03.012
8. Kuramoto Y. Chemical Oscillations, Waves, and Turbulence [Internet]. Vol. 19. Berlin, Heidelberg: Springer Berlin Heidelberg; 1984 [cited 2025 Nov 24]. (Haken H, editor. Springer Series in Synergetics). Available from: http://link.springer.com/10.1007/978-3-642-69689-3 doi:10.1007/978-3-642-69689-3
9. Yeo BTT, Krienen FM, Sepulcre J, Sabuncu MR, Lashkari D, Hollinshead M, et al. The organization of the human cerebral cortex estimated by intrinsic functional connectivity. Journal of Neurophysiology. 2011 Sep;106(3):1125. doi:10.1152/jn.00338.2011 PubMed PMID: 21653723.
10. Sydnor VJ, Larsen B, Bassett DS, Alexander-Bloch A, Fair DA, Liston C, et al. Neurodevelopment of the association cortices: Patterns, mechanisms, and implications for psychopathology. Neuron. 2021 Sep 15;109(18):2820–46. doi:10.1016/j.neuron.2021.06.016 PubMed PMID: 34270921.
11. Margulies DS, Ghosh SS, Goulas A, Falkiewicz M, Huntenburg JM, Langs G, et al. Situating the default-mode network along a principal gradient of macroscale cortical organization. Proceedings of the National Academy of Sciences. 2016 Nov;113(44):12574–9. doi:10.1073/pnas.1608282113
12. Tsyporin J, Zhang M, Qi C, Segal A, Finn T, Kim H, et al. Competing Programs Shape Cortical Sensorimotor-Association Axis Development [Internet]. bioRxiv; 2025 [cited 2025 Jul 29]. p. 2025.06.26.660775. Available from:

https://www.biorxiv.org/content/10.1101/2025.06.26.660775v1 doi:10.1101/2025.06.26.660775
13. Bernhardt BC, Valk SL, Hong SJ, Soulières I, Mottron L. Autism-related shifts in the brain's information processing hierarchy. Trends in Cognitive Sciences. 2025 Jun 16. doi:10.1016/j.tics.2025.04.008
14. Lombardo MV, Mandelli V. Rethinking Our Concepts and Assumptions About Autism. Front Psychiatry. 2022 Jun 3;13:903489. doi:10.3389/fpsyt.2022.903489 PubMed PMID: 35722549; PubMed Central PMCID: PMC9203718.
15. Gandal MJ, Haney JR, Wamsley B, Yap CX, Parhami S, Emani PS, et al. Broad transcriptomic dysregulation occurs across the cerebral cortex in ASD. Nature. 2022;611(7936):532–9. doi:10.1038/s41586-022-05377-7 PubMed PMID: 36323788; PubMed Central PMCID: PMC9668748.
16. Alexander-Bloch AF, Shou H, Liu S, Satterthwaite TD, Glahn DC, Shinohara RT, et al. On testing for spatial correspondence between maps of human brain structure and function. NeuroImage. 2018 Sep 1;178:540–51. doi:10.1016/j.neuroimage.2018.05.070
17. Markello RD, Misic B. Comparing spatial null models for brain maps. NeuroImage. 2021 Aug 1;236:118052. doi:10.1016/j.neuroimage.2021.118052
